# Laparoscopic gynecological surgery in an adult woman with Becker muscular dystrophy performed with sevoflurane with cisatracurium anesthesia: A case report

**DOI:** 10.1097/MD.0000000000019733

**Published:** 2020-04-17

**Authors:** Shi-Yao Zhou, Dan Wang, Chang Liu, Shi Zhang, Bao-Lei Shan, Hai-Chun Ma

**Affiliations:** a Department of Anesthesiology; b Department of Orthopaedic Surgery, The First Hospital of Jilin University, Changchun, Jilin, China.

**Keywords:** Becker muscular dystrophy, bilateral adnexectomy, cisatracurium besilate, laparoscopic hysterectomy, sevoflurane

## Abstract

**Rationale:**

Becker muscular dystrophy (BMD) and Duchenne muscular dystrophy (DMD) are progressive neuromuscular disorders caused by mutations in the dystrophin gene. The management of anesthesia in patients with BMD is complicated because they are highly sensitive to the conventional anesthetics such as volatile anesthetics and muscle relaxants. It is reported that anesthesia in patients with DMD is associated with several complications. However, a few case reports have been published on adult patients with BMD undergoing surgery with general anesthesia. Reports indicate that children with BMD may experience some serious complications with flurane-inhaled anesthesia. However, no study has yet shown that the use of flurane-induced anesthesia in adults with DMD carries high risks.

**Patient concerns:**

We describe a 56-year-old woman with BMD who was scheduled for laparoscopic hysterectomy and bilateral adnexectomy under general anesthesia due to a mass in the uterus. The patient was diagnosed with BMD 20 years back and reported that during this period, she was able to walk slowly with help during her daily life. Additionally, she also had a history of hypertension since 4 years and type 2 diabetes mellitus since 2 years.

**Diagnosis:**

The patient was postmenopausal and presented with abnormal uterine bleeding and elevated CA125. Abdominal ultrasonography revealed diffuse enlargement of the uterus and hypoechoic internal echoes. These findings were suggestive of diffuse adenomyosis with multiple uterine leiomyomas, which would have adverse effects later in her life. Therefore, the patient required surgery to address the symptoms and further confirm the diagnosis. The final diagnosis was confirmed by histopathological analysis.

**Interventions:**

The patient was scheduled for laparoscopic hysterectomy and bilateral adnexectomy. Anesthesia was induced and maintained by a combination of intravenous and inhalation anesthetic agents, particularly cisatracurium besilate and inhaled. sevoflurane.

**Outcomes:**

The duration of anesthesia and postoperative period were uneventful. At the end of the operation, the patient had normal vital signs and was fully conscious. The patient was followed up for 8 months and no complications were noted during this period.

**Lessons:**

The combination of sevoflurane and cisatracurium besilate is a safe and effective method for the anesthetic management of adult patients with BMD scheduled for laparoscopic gynecological surgery. On the other hand, it is important to be aware of even rare complications of procedures, so that necessary precautions can be undertaken. Further investigations are necessary to determine the safe dosage of volatile anesthetics specifically for this clinical scenario so that anesthesiologists can use this combination method more accurately and precisely.

## 1 Introduction

In recent times, laparoscopic hysterectomy is often performed more frequently in clinical practice than abdominal hysterectomy. The widespread popularity of the laparoscopic approach can be attributed to its advantages of small incisions, low rate of complications, and reduced duration of hospitalization and recovery.^[1]^ Becker muscular dystrophy (BMD) and Duchenne muscular dystrophy (DMD) are hereditary muscle disorders that involve progressive neuromuscular changes caused by mutations of the dystrophin gene.

Patients with BMD as well as those with the scapular, sacral, ocular, and distal types of muscular dystrophy have longer survival and milder disease presentation as compared with those with DMD. The clinical features of BMD include progressive weakness of the proximal part of the lower limb and pelvic muscles, followed by muscular weakness of the upper limb, shoulder, and neck.^[2]^ With respect to surgical procedures in patients with muscular dystrophy, anesthesia induction is associated with various perioperative complications such as rhabdomyolysis, hyperkalemic cardiac arrest, and intra- and postoperative hyperthermia.^[3–8]^

As per the current understanding, succinylcholine is considered to be contraindicated in such conditions, and there is no consensus on whether volatile anesthetics should be used.^[9]^ Cisatracurium besilate is a non-depolarizing muscle relaxant that exerts its effects by binding to cholinergic receptors on the motor endplate, thereby antagonizing the action of acetylcholine and inducing a competitive neuromuscular block. The action of cisatracurium besilate does not require a dose-dependent histamine release, and it has been shown to be safer than other neuromuscular blocking agents such as atracurium and vecuronium.^[10]^ Therefore, it could be considered as being relatively safe for patients with BMD.

Further, the perioperative complications in patients with muscular dystrophy may be associated with the use of volatile and non-volatile anesthetics.^[11]^

No definitive curative therapies have been identified for muscular dystrophy. Since patients with dystrophy present with a wide range of symptoms, the associated health concerns are different for each individual patient. The advantages of the use of volatile anesthetics cannot be disregarded in patients, especially in the case of adults with BMD, although the use of volatile anesthetics in children with DMD has been reported to cause a few adverse effects.

In this paper, we report the successful completion of elective laparoscopic hysterectomy and bilateral adnexectomy in a 56-year-old woman with BMD.

## 2 Case report

The patient (age: 56 years; weight: 48 kg) presented with a mass in the uterus for which laparoscopic hysterectomy and bilateral adnexectomy under general anesthesia were recommended. She had been diagnosed with BMD at the age of 36 on the basis of her muscle biopsy findings and was able to walk slowly with help. Physical examination revealed that she was in good general condition. Her limbs showed hypertrophy, with slight bilateral calf hypertrophy. Further, the gluteal muscles showed thinning. Her gait showed typical signs of muscle dystrophy, including toe walking, waddling, and leaning the body towards the other side to balance the center of gravity. On examination, both lower limbs showed decreased muscle power against resistance due to knee retractions and clubfoot. She had a history of hypertension for 4 years, with blood pressure raising up to 160/100 mmHg, and type 2 diabetes mellitus for 2 years. Preoperative evaluation revealed elevated levels of serum creatine kinase (CK) and glucose.

Before the start of the operation, standard monitoring was initiated, including electrocardiography, pulse oximetry, and noninvasive arterial blood pressure measurement. Train-of-four (TOF) stimuli were applied to the ulnar nerve to monitor neuromuscular blockade (NMB) using an electromyographic neuromuscular transmission module (M-NMT Module; Datex Ohmeda Inc, Helsinki, Finland). A TOF ratio (%) of 100% was considered as a reversal of the NMB.

The patient was placed in the supine position with an anesthesia face mask that administered oxygen at the rate of 6 L/min for 3 minutes. For the induction of anesthesia, 2 mg of midazolam was administered with 16 mg etomidate and 20 μg sufentanil. Anesthesia was maintained by an infusion of 75 to 100 μg/kg/min propofol and 0.05 to 2 μg/kg/min remifentanil administered using a syringe pump until termination of the operation, which lasted 90 minutes. The TOF ratio (%) at the initial measurement was 90%, which reduced to 4 when the patient was administered a bolus injection of 5 mg cisatracurium besilate. After the completion of endotracheal intubation, the lungs were inflated by the administration of a 1:2 mixture of oxygen and air; modified Allen test was performed to invasively monitor blood pressure and then the left radial artery was cannulated. Further, 10 μg of sufentanil was administered 5 minutes before the start of surgery in order to avoid a stress response. The operation was started half an hour after intubation; to maintain anesthesia, 2% sevoflurane was administered 35 minutes after anesthesia induction because the TOF count was 3, while the TNF ratio was 30%. The electromyography showed a tendency to increase over 50. The duration of the operation was 90 minutes, while anesthesia was maintained for 102 minutes. Sixty-five minutes after intubation, the patient was administered 5 μg sufentanil and 30 mg ketorolac for postoperative analgesia. At the time of completion of the procedure the TOF ratio was 99%, which indicated that there was nearly no NMB. The administration of sevoflurane did not cause any clinically relevant changes in arterial blood pressure or heart rate as compared with the baseline levels. Extubation was safely performed after the surgery, and the patient was shifted to the post-anesthesia recovery unit, where she was monitored for apnea symptoms. Urine analysis was performed to check for myoglobinuria induced by rhabdomyolysis. The patient did not develop any adverse effects, such as hyperthermia and hypersensitivity, after the introduction of sevoflurane. Recovery from anesthesia was uneventful, and the patient was transferred to the postoperative recovery ward, where she did not show any signs of residual NMB or recurarization. An hour later, the patient was shifted to the ward. The patient was followed up for 8 months, and no complications were noted during this period.

Written informed consent was obtained from the patient for the publication of this report.

## 3 Discussion

BMD is an inherited, congenital muscle disorder that differs from DMD in terms of slower progress and less severity. The onset of DMD occurs in early childhood and initially manifests with difficulties in running and then in climbing stairs. The course of muscle wasting in BMD is similar to that in DMD; however, the former condition is more benign than the latter. Nevertheless, both conditions can eventually lead to respiratory and cardiac insufficiency. BMD is a rare condition affecting very young men.^[12]^ To our knowledge, only 1 case of an adult with BMD undergoing anesthesia induction has been reported thus far, and no new cases have been recorded from our country.^[12]^ This is because patients with muscular dystrophy are known to be susceptible to more sensible to the anesthetics, such as volatile and relaxant agents. Poole et al^[13]^ reported unpredictable cardiac arrest after anesthesia with isoflurane; patient underwent cardiopulmonary resuscitation (CPR) and recovered without any adverse events on functional or cognitive levels. One survey stated that using volatile agents for patients with BMD is acceptable only if they are closely monitored with vital signs.^[14]^ Milne and Rosales^[15]^ reported the case of a child with BMD undergoing spinal fusion surgery for scoliosis under general anesthesia. There were no anesthetic complications during operation, and the patient's vital signs were within normal limits. Therefore, it has been challenging to accomplish the anesthesia work for patients with BMD and DMD.

An important concern in BMD is the prevention of hyperkalemia as well as malignant hyperthermia. In our case, we were able to complete the surgical procedure without any of these complications. Overall, total intravenous anesthesia induced by the administration of new, short-acting anesthetics and non-depolarizing muscle relaxants, and rarely, recent volatile anesthetics, such as desflurane and sevoflurane, can promote recovery and result in reducing postoperative complications.^[12]^ The use of succinylcholine with sevoflurane for the induction of anesthesia has been reported to cause have severe complications such as cardiac arrest; therefore, some investigators advise against the usage of both the drugs. However, no systematic studies have yet provided sufficient evidence against the use of sevoflurane. The combined approach of intravenous–inhalation anesthesia is considered a safe and effective method that ensures successful completion of operative procedures. The concomitant use of sevoflurane allows for a reduction in the dose of the intravenously administered anesthetic, and a synergistic effect is obtained. Thus, the combined use of sevoflurane and cisatracurium besilate allows for muscle relaxation and helps regulate the depth of anesthesia for the surgery, without any increase in the risk of undesirable complications such as the hyperkalemia and malignant hyperthermia, which are common when either of these agents is used individually. Further, the effective dosage (ED) of cisatracurium besilate should be adjusted from triple ED95 to single ED95 since patients with BMD are more sensitive to non-depolarizing and depolarizing relaxants.

It is important to be aware of even rare complications of procedures, so that necessary precautions can be undertaken. However, more research is warranted about the different pharmacological effects of anesthetics in children and adults with DMD or BMD requiring surgery. Older patients with BMD may be less subject to rhabdomyolysis on exposure to inhaled anesthetics because they have less muscle mass owing to disease progression.^[16]^ The minimal use of non-depolarizing relaxants such as cisatracurium besilate would improve the safety of the procedure and facilitate intubation, thereby enabling efficient airway control.

## 4 Conclusions

The current opinion is that the use of volatile anesthetics and relaxants should be generally avoided when considering general anesthesia in children with BMD or DMD. Further, there are no definite recommendations for general or regional anesthesia in adult patients. From our experience in this case, we believe that the combination of intravenous–inhalation anesthesia induced and maintained by sevoflurane and cisatracurium besilate may be used in successfully completing surgical procedures in adult patients with muscular dystrophy.

## Author contributions

**Conceptualization:** Shiyao Zhou, Dan Wang, Bao-Lei Shan, Hai-Chun Ma.

**Data curation:** Shiyao Zhou, Dan Wang, Chang Liu.

**Funding acquisition:** Hai-Chun Ma.

**Methodology:** Dan Wang.

**Project administration:** Hai-Chun Ma.

**Resources:** Dan Wang.

**Supervision:** Dan Wang, Bao-Lei Shan, Chang Liu, Hai-Chun Ma.

**Validation:** Dan Wang, Bao-Lei Shan, Chang Liu, Shi Zhang, Hai-Chun Ma.

**Visualization:** Shiyao Zhou, Dan Wang, Shi Zhang.

**Writing – original draft:** Shiyao Zhou, Dan Wang, Bao-Lei Shan.

**Writing – review & editing:** Shiyao Zhou, Dan Wang, Bao-Lei Shan, Chang Liu, Shi Zhang, Hai-Chun Ma.
